# Supplementary material for: PanKB: An interactive microbial pangenome knowledgebase for research, biotechnological innovation, and knowledge mining
Source: Nucleic Acids Res. 2024 Nov 22;53(D1):D806–18. doi: 10.1093/nar/gkae1042 (PMC11701538; doi:10.1093/nar/gkae1042)
Supplement: gkae1042_Supplemental_Files [file gkae1042_supplemental_files.zip › Supplementary_Materials.pdf]

Supplementary Materials

Supplementary Figure

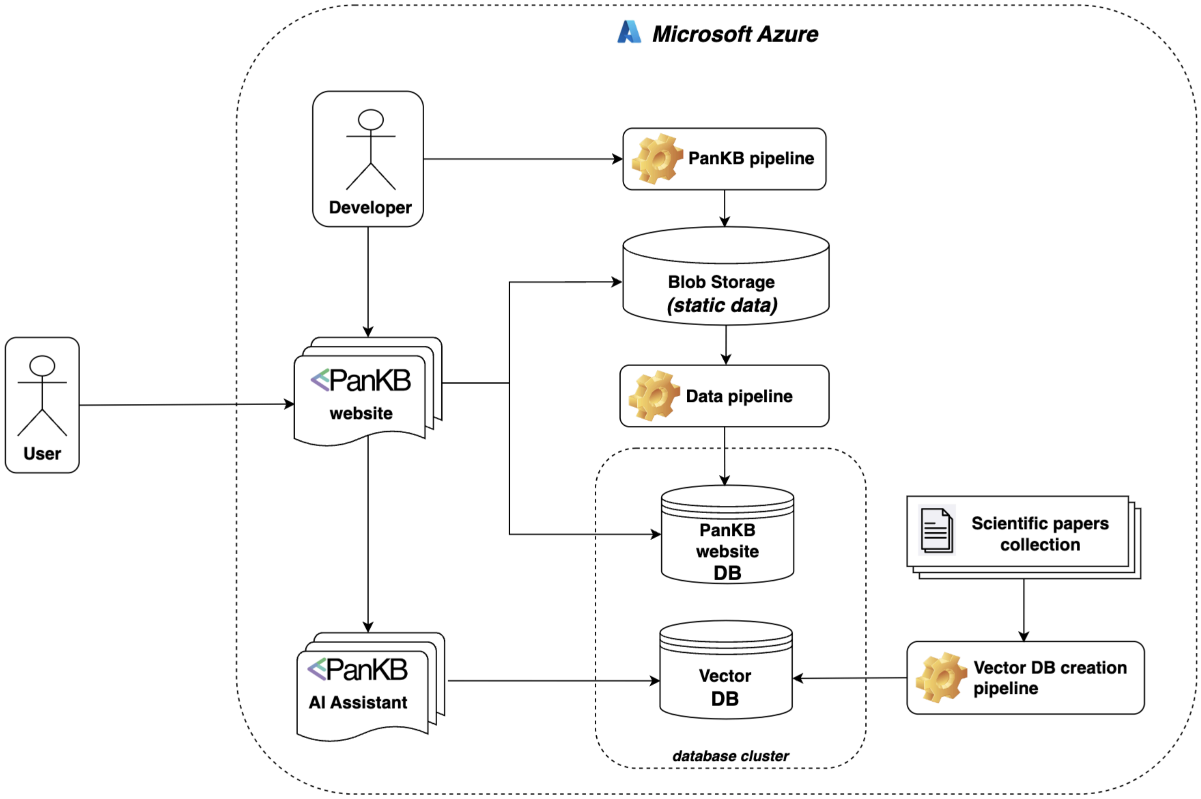

Figure S1. The PanKB project comprises the website, AI Assistant web application, three data processing pipelines, two databases and one blob storage. The data used by the PanKB website are stored in Blob Storage and the PanKB website DB. The Vector DB is a knowledge base for the AI Assistant application. All the infrastructure components are cloud-based and hosted on Microsoft Azure.

Supplementary Table

Table_S1.xlsx
